# Improving the Quality of Adolescent and Youth-Friendly Health Services Through Integrated Supportive Supervision in Four Nigerian States

**DOI:** 10.9745/GHSP-D-22-00169

**Published:** 2024-05-21

**Authors:** Dorcas Akila, Akinola Oluwasegun, Krishna Bose, Olukunle Omotoso, Adewale Adefila, Lisa Mwaikambo

**Affiliations:** a The Challenge Initiative, Nigeria Hub, Johns Hopkins Center for Communication Programs, Abuja, Nigeria.; b The Challenge Initiative, Johns Hopkins Center for Communication Programs, Baltimore, MD, USA.

## Abstract

**Background:**

Although the unique sexual and reproductive health needs of adolescents and youth (AY) are widely recognized, the challenge remains how to integrate adolescent- and youth-friendly health services (AYFHS) effectively within a systems-based approach that is both feasible and scalable. This article provides preliminary evidence from 4 Nigerian states that sought to overcome this challenge by implementing capacity-strengthening approaches centered around a shortened quality assurance (QA) tool that has become part of the state health system’s routine supportive supervision process and follow-up quality improvement (QI) activities.

**Methods:**

A shortened QA tool was administered to assess and track the performance of 130 high-volume health facilities across 5 domains to serve its AY population with quality contraceptive services. Facility-based providers (N=198) received training on adolescent and youth sexual and reproductive health, AYFHS, and long-acting reversible contraceptive methods. To corroborate checklist findings, we conducted exit interviews with 754 clients (aged 15–24 years) who accessed contraceptive services from the facilities that met the World Health Organization’s minimum standards for quality AYFHS.

**Results:**

In the 4 states, the QA tool was applied at baseline and 2 rounds, accompanied by QI capacity strengthening after each round. At baseline, only 12% of the 130 facilities in the 4 states scored met the minimum quality standards for AYFHS. After 2 rounds, 88% of the facilities met the minimum standards. AY client volume increased over this same period. All 4 states showed great improvements; however, the achievements varied by state. The exit interview feedback supported client satisfaction with the services provided to AY.

**Conclusion:**

Integrating QA followed by QI within Nigeria’s family planning supportive supervision system is not only feasible but also impacts the quality of AYFHS and contraceptive uptake by clients aged 15–24 years.

## Abstract (precis)

Integrating quality assurance in Nigeria’s family planning supportive supervision system improved the quality of adolescent- and youth-friendly health services and contraceptive uptake by clients aged 15–24 years.

## Teaser

**Key Findings:**

- Adapting a regional adolescent- and youth-friendly family planning supportive supervision tool within an existing government health supportive supervision system, accompanied by provider capacity strengthening and coaching, can help improve the quality of adolescent- and youth-friendly health services (AYFHS) provided.
- This facility-level AYFHS intervention, combined with community-level demand-generating activities and youth social mobilization efforts, can facilitate an increased demand and uptake of modern contraceptive services by adolescents and youth.

**Key Implications:**

- Effective policy-level support at the national, state, and facility levels is critical to improving AYFHS quality and contraceptive uptake among adolescents and youth.
- Application of the supportive supervision tool and its accompanying processes should be built into existing supportive supervision systems to ensure ongoing behavior change among providers and system-level improvements to ensure adolescent-responsive contraceptive services that are available long term.

## BACKGROUND

According to the United Nations’ population projections for 2020, about 43% and 19% of the Nigerian population comprised children (younger than age 14 years) and youth (aged 15–24 years), respectively.^1^ With such a large cohort of youth in the sixth most populous country in the world, there is a need to ensure their adequate access to quality sexual and reproductive health (SRH) services, including contraceptives, as they go through puberty and transition into young adulthood. The challenge faced not only in Nigeria but also throughout sub-Saharan Africa is that existing SRH services need to be capacitated to effectively address the unique needs of adolescents and youth (AY) to improve service quality and utilization by them.^2^^–^^6^ AY often forego SRH services due to a variety of concerns and barriers, including a lack of awareness on where to obtain services, worries about stigma, embarrassment, confidentiality and privacy concerns, parental consent requirements, cost of services, distance, and negative provider attitudes.^7^^–^^9^ Furthermore, existing SRH services are configured for adults and often served by adults who are not sensitive to the SRH needs of AY.^10^ This lack of sufficient availability of quality health services focused on AY needs, combined with the relatively low demand and utilization by this cohort, contributes to unprotected sex and unintended pregnancies among AY.^11^

The World Health Organization (WHO) recommends adolescent- and youth-friendly health services (AYFHS) as one of the evidence-based interventions to address several of these barriers. By creating a health-enabling social and health systems environment, such services are more accessible, acceptable, equitable, appropriate, and effective for young people.^12^ The range of services and commodities provided include SRH education, counseling, and referrals for contraceptives, including condoms, HIV counseling and testing, and STI screening and treatment.^13^^–^^15^ ^5^ It is also of note that the quality of contraceptive service provision has been shown to be a key determinant in the uptake and consistent use of contraceptives.^16^

By creating a health-enabling social and health systems environment, AYFHS are more accessible, acceptable, equitable, appropriate, and effective for young people.

However, the challenge remains to adapt existing health services supportive supervision structures to provide quality AYFHS and contraceptive services with ongoing quality improvement (QI) activities.^17^^–^^20^ In 2015, WHO published a guide for assessing the quality of health care services for adolescents based on implementing a standards-driven approach to QI, representing global standards based on a health systems framework and informed by country-level best practices. The guide included implementation steps and tools for measuring the quality and coverage of such services.^21^ These tools are rigorous, comprehensive, and well-suited for evaluating the quality of AYFHS. For ongoing monitoring in support of QI programming efforts, the WHO’s Regional Office for South-East Asia (SEARO) adapted an AYFHS checklist (Box),^22^ which is organized into 6 domains that include 17 criteria that link to at least 1 of the 8 global AYFHS standards that The Challenge Initiative (TCI)^23^ adapted, as well as to the Nigeria National Standards and Minimum Service Package for Adolescent & Youth Friendly Health Services.^12^^,^^15^

BOX The World Health Organization Regional Office for South-East Asia Adolescent and Youth-Friendly Health Service Checklist Domains

1. Provision of servicesInformation/advice on sexual and reproductive health concerns is provided. It could include 1 or more of the following for each client.
  - Sexually transmitted infections/reproductive tract infections treatment
  - Provision of condoms free of cost
  - Provision of contraceptives including emergency contraceptive pills free of cost
  - Provision of referral services
2. Facility check
  - Signboard with clinic information and policy on confidentiality on display
  - Consultation/examination room ensures privacy
  - Records of adolescent clients kept under lock and key
  - Clean and functional toilets available
3. Capacity of providers
  - Received trainings on adolescent- and youth-friendly health services (AYFHS)/adolescent and youth sexual and reproductive health (AYSRH)
  - Have confidence in dealing with adolescent clients
4. Demand creation for services
  - Educational materials on AYSRH are displayed and available for adolescents either to read or take home
  - No stock-outs of information, education, and communication materials
  - Dissemination of AYFHS/ASRH information to community members
5. Feedback from adolescent clients
  - Service providers were friendly, respectful, and nonjudgmental toward adolescent clients
  - Are you satisfied with the services that you received here today?
6. Data on client visits and outreach services in the last quarter
  - Number of adolescents attended routine clinics/outpatient department

## TCI’S AYSRH PROGRAM

TCI had already supported the FP program in 10 states in Nigeria. In June 2018, after documenting the lessons learned from the successful implementation of high-impact family planning (FP) interventions in several countries, including Nigeria, TCI received additional funding from the Bill & Melinda Gates Foundation to sharpen its focus on improving contraceptive access for AY aged 15 to 24 years—within the larger cohort of women of reproductive age (aged 15 to 49 years), who were already supported by its FP program. This funding allowed TCI to provide technical and program support for married and unmarried AY aged 15 to 24 years in 4 TCI-supported states in Nigeria by mobilizing demand for and improving the quality of AYFHS.^24^

In September 2018, TCI Nigeria’s adolescent and youth sexual and reproductive health (AYSRH) program rolled out in Niger and Ogun states, where TCI had already supported the states’ FP program. In November 2019, the program expanded to Plateau and Edo, the only state that TCI was not already supporting with an FP program before commencing support for TCI AYSRH programming.

TCI’s demand-driven approach ensures that local governments that choose to partner with TCI pledge their own financial and human resources and political commitment. The State Ministry of Health (SMOH) usually serves as the coordinating platform for all health programming and operates more from the state capital to ensure policy formulation, development of budgeted annual operational plans, and funding. The State Primary Health Care Development Agency (SPHCDA) operates at the grassroots level to facilitate the implementation of those health interventions in the approved and budgeted annual operational plans. TCI provided technical support to the SMOH and the SPHCDA and funding from The Challenge Fund to adapt, implement, and monitor state-led proven FP and AYSRH interventions in poor urban settlements, integrating the interventions into the annual operational plans when possible. This support included improving the delivery of AYFHS within existing high-volume facilities using simplified and standardized quality assurance (QA) tools. Another critical complementary component of TCI’s support was providing community-level social mobilization support with demand-generation activities, which have been shown to be linked to increased uptake of modern contraceptive methods in urban Nigeria by the Nigeria Urban Reproductive Health Initiative, funded by the Bill & Melinda Gates Foundation.^25^

TCI supported the state governments in improving the delivery of AYFHS within existing high-volume facilities using standardized QA tools.

The WHO SEARO checklist^22^ and Nigeria’s National Standards & Minimum Service Package for Adolescent & Youth Friendly Health Services^15^ were integral documents used in updating the already existing family planning supportive supervision (FPSS) tool that the Nigerian government had previously endorsed. To ensure that the quality of FP service delivery aligned with the national FP service protocol and facilitate improvement in the quality of service delivery, SMOH and SPHCDA staff conducted supportive supervision activities using the FPSS QA tool at the health facilities.

This program case study aims to answer the following 2 programmatic questions.

1. What are the effects of enhancing the supportive supervision system of the SMOH and SPHCDA through the implementation of a QA checklist on improving the quality of AYFHS at scale?
2. Does improving the quality of AYFHS influence the demand and uptake of contraceptives by AY?

## IMPLEMENTATION OF THE AYFPSS QA TOOL

TCI supported the SMOH Adolescent Health and Development unit and the SPHCDA to identify and select 130 high-volume sites (HVS) (primary health centers) across 25 urban local government areas (LGAs) in Edo, Niger, Ogun, and Plateau states to implement AYFHS QI interventions. These HVS facilities had the highest client volume and high caseloads for antenatal care, labor and delivery, childhood routine immunizations, and FP services.

The interventions implemented included enhancing supportive supervision using a modified FPSS QA checklist to provide facility-level coaching and whole-site orientation, strengthening provider capacity in AYFHS at facilities through training and mentoring, and strengthening AY demand for contraceptive services.

### Enhance Supportive Supervision Visits Using Modified FPSS Checklist

The state’s supportive supervision visits at health facilities were conducted typically quarterly or bi-annually, depending on the availability of state funds and capacity to conduct the exercise. The average interval between baseline and round 1 supportive supervision visits was 10 months to slightly more than 12 months and 5 to 7 months between rounds 1 and 2. During these visits, SMOH and SPHCDA staff used a checklist to monitor service quality. However, not all states used the same checklist. In Ogun, Niger, and Plateau States, where TCI had been supporting the government’s FP program, state officials used the FPSS checklist. In Edo, where TCI had not been supporting the government’s FP program, state officials used the integrated supportive supervision checklist, which only includes 4 items related to FP service delivery. TCI worked with the states to adapt and incorporate AYFHS into the FPSS checklist and introduced a modified checklist, referred to as the adolescent and youth family planning supportive supervision checklist (AYFPSS), into routine supportive supervisory visits of the 130 HVS across the 4 states.

To monitor FP service quality at health facilities, SMOH and SPHCDA staff used a supportive supervision checklist; but all 4 states did not use the same checklist.

At baseline, Ogun, Niger, and Plateau states showed considerable health system readiness in FPSS. Political leadership in those states had expressed a willingness to increase access to contraceptives for AY aged 15–24 years, as demonstrated by public statements and commitment of local government funds to support FP and AYSRH programming; to train providers in FP; and to take steps toward ensuring a functional supply chain system, health management information system (HMIS), and supportive supervision structure.^26^ This health system readiness was the result of actions taken by the local governments in Ogun, Niger, and Plateau and supported by implementing partners. In contrast, at baseline, Edo state lacked health system readiness. Trained FP and AYFHS providers were not driving supportive supervision, costed annual operational plans did not include AYSRH nor integrated supportive supervision, and no implementing partners supported the SMOH’s FP and AYSRH programs.

### Strengthen Capacity Transfer

Each HVS documented capacity gaps in AYFHS in all 3 rounds of the AYFPSS. To address these gaps and improve the quality of service in the facilities, government staff worked with facility staff to develop performance improvement plans (PIPs). The PIPs included a variety of capacity transfer approaches, such as coaching, mentorship, on-the-job training, and advocacy visits, as well as engagement with policymakers and health managers before the next supportive supervision visit to secure funding commitments to address issues of stock-outs, procurement of required equipment, and training support. To facilitate ownership of addressing the gaps, key stakeholders discussed PIPs during contraceptive technology update meetings organized by SMOH and SPHCDA.

Findings from the first round of application of the AYFPSS found that most facilities lacked trained FP providers and many providers expressed a bias in delivering contraceptive services to AY. As a result, most PIPs called for provider trainings. Using the National Training Manual for the Health & Development of Adolescent and Young People in Nigeria, TCI, in conjunction with the SMOH and SPHCDA, trained 198 facility-based providers on FP, AYFHS, long-acting reversible contraceptives, and interpersonal, communication, and counseling skills. State and LGA supportive supervision teams provided facility-level coaching during routine supportive supervision visits to ensure quality service provision across the 130 facilities. In addition to supportive supervision, PIP development, and facility-level coaching, TCI supported on-site whole-site orientation of all facility staff with a focus on 5 additional AYFHS topics and introduced the value of QI team meetings to strengthen the connection between the facility-based staff and the community it serves.

### Facilitate Uptake of Contraceptive Services by AY

To complement the QI interventions for service delivery and strengthen the demand for services, TCI supported the SMOH and SPHCDA in developing state-specific social and behavior change (SBC) based on findings from a household survey conducted in the 4 states with 7,011 AYs distributed to the 130 HVS.^15^^,^^27^^,^^28^ In addition, TCI supported the states to recruit and train youth social mobilizers, who disseminated the SBC materials and messages throughout the HVS catchment areas to generate demand for contraceptive services and ensure that AY knew where such services were available to them. Social mobilizers conducted neighborhood campaigns and referred AY to the 130 facilities for FP services using a referral card called the “Go-Card.” To facilitate tracking of completed referrals, the triplicate Go-Card included the contact information of the social mobilizer who made the referral and the contact information and age range of the adolescent or youth referred to services. During demand-generation activities, social mobilizers also used and distributed leaflets, such as Z-cards (Supplement). These leaflets contained information about issues concerning AY, brief descriptions of contraceptive methods and life skills important to AY, and a list of health facilities within their communities. Social mobilizers distributed these leaflets to AY during social mobilization activities in the communities to increase awareness and decision-making about contraceptive use.

TCI supported states to recruit and train youth social mobilizers to disseminate SBC materials to generate demand for and raise awareness of contraceptive services.

TCI also developed a program monitoring information system to track and report contraceptive uptake by AY in the 130 facilities using the redeemed Go-Cards to measure uptake since Nigeria’s HMIS does not report on data disaggregated by age and method. However, these data are reported in the facility registers. As a result, TCI supported the SPHCDA staff in retrieving age disaggregated data for individuals aged 15–19 years, 20–24 years, and older than 25 years from the facility registers and inputting it into the program monitoring information system.

## METHODS

This program case study employed 2 quantitative datasets, captured from the facility service provider’s perspective and the AY client’s perspective.

### Facility Service Provider’s Perspective

The AYFPSS checklist was administered at baseline in November 2019 in Ogun and Niger and in June 2020 in Plateau and Edo. Round 1 was administered in November 2020 in the 3 states and in February 2021 in Edo. Round 2 administration was in June 2021 in 3 states and in August 2021 for Edo. This checklist assessed and monitored the HVS’ ability to deliver quality contraceptive services to their AY populations. The data collection rounds took place at different time periods, depending on when TCI began its partnership with the state. The AYFPSS checklist measures 5 core domains using the following point system: service delivery (6 points), facility verification (4 points), service provider capacity (3 points), service demand creation (4 points), and data records (2 points) (Supplement). Aggregate assessment scores ranging from 5 to 19 were classified by colors: red (5–8 points) for facilities that did not meet the minimum quality standards for AYFHS, yellow (9–13 points) for those that met some standards but not all, and green (14–19 points) for facilities that met all the minimum quality standards.

Between 2019 and 2021, assessments were conducted in the 130 HVS: 30 facilities in Edo state, 28 in Niger, 47 in Ogun, and 25 in Plateau.

### Clients’ Perspectives

#### Target Population and Sample Size

To learn from the AY client’s perspective about the quality of services, the study team invited AY contraceptive users aged 15–24 years to participate in client exit interviews. Clients to be interviewed were selected from the 114 facilities that met all the minimum quality standards, scoring green after round 2 of the assessment. The client interviews helped to triangulate the findings from the AYFPSS, strengthening our understanding of clients’ experiences at the health facilities that reported improvements in quality AYFHS provision. Interview questions included perceptions of service quality, facility characteristics, wait time, and perceived level of satisfaction with client-provider interaction.

A total of 754 clients aged 15–24 years across the 4 TCI states participated in the study: 256 respondents were adolescents aged 15–19 years and 498 were youth aged 20–24 years. To determine eligibility for the study, the study team screened potential participants to match the criteria. Of the 754, 739 clients gave their verbal informed consent to be interviewed. The interviews were conducted in English, Hausa, and Pidgin languages to ensure participants' comfort with the language of the interview. These interviews were not recorded, as they were conducted in person.

### Data Collection/Analysis

Quantitative data were collected from the respondents across the 114 green facilities via an open-source mobile data collection platform, Open Data Kit (ODK), for 2 weeks in September 2021. Data collected included respondents’ sociodemographic characteristics, perceptions of service quality, facility characteristics, wait time, and perceived level of satisfaction with client-provider interaction. Data were checked routinely for consistency and QA; where there was an outlier(s), the data teams were notified, and data cleaning commenced. Data were exported to STATA version 14 for analysis. Results were analyzed using descriptive and inferential statistics.

### Ethical Approval

All study materials, including the study protocol and tools, used across the 4 states (Edo, Ogun, Niger, and Plateau) received ethical approval from each state’s respective health research ethics committee to conduct the study.

## RESULTS

The results from the AYFPSS assessment were analyzed for 130 HVS in the 4 TCI-supported AYSRH states (Table 1). Only 12% of the facilities assessed at the baseline met the minimum quality standard (i.e., green). When compared to the baseline, scores from round 1 and round 2 assessments showed that 63% and 88%, respectively, of the facilities met the minimum quality standard (i.e., green). Edo state showed great improvements as more than 70% of its facilities met the minimum quality standard at round 2 from a baseline of 0%.

**TABLE 1.** Facilities Meeting AYFHS Minimum Standards in Four Nigerian States

		**Baseline, %**	**Round 1, %**	**Round 2, %**
All 4 states (N=130)	Red	35	5	0
	Yellow	53	32	12
	Green	12	63	88
Ogun (n=47)	Red	13	0	0
	Yellow	87	43	15
	Green	0	57	85
Plateau (n=25)	Red	52	0	0
	Yellow	48	24	0
	Green	0	76	100
Niger (n=28)	Red	0	0	0
	Yellow	54	0	0
	Green	46	100	100
Edo (n=30)	Red	73	20	0
	Yellow	27	57	30
	Green	0	23	70

Abbreviation: AYFHS, adolescent- and youth-friendly health services.

After 2 rounds of assessment, 88% of all health facilities met the minimum quality standard, compared to 12% at baseline.

All facilities in Niger and Plateau met the minimum standard of quality for AYFHS after the 2 rounds of the AYFPSS assessment were conducted, while 70% and 85% of facilities in Edo and Ogun, respectively, met the quality standards for AYFHS. At baseline, Niger state already had 46% of its facilities scoring green and meeting the minimum standards of quality AYFHS.

Table 2 presents the FP client volume by AY clients in the 4 states across the different rounds of the assessment. For Ogun and Niger states, AY clients in round 1 increased by more than 4 times when compared with client volume at baseline, depicting a 4-fold increase. After round 2, there was a more than 5-fold increase in client volume compared to baseline.

**TABLE 2.** Family Planning Client Volume by AY Clients at the 130 Facilities After AYFPSS Assessments and Their Percentage Change Across Four Nigerian States

	**Baseline, No.**	**Round 1, No.**	**Round 2, No.**	**Change Between Baseline and Round 1, %**	**Change Between Baseline and Round 2, %**
Ogun	1,456	7,355	8,967	405	516
Niger	2,319	12,024	14,748	418	536
Plateau	1,315	2,112	3,849	61	193
Edo	1,255	1,514	1,936	21	54

Abbreviations: AY, adolescents and youth; AYFPSS, adolescent and youth family planning supportive supervision.

At baseline, between December 2017 to December 2018, about 2,000 clients aged 15–24 years were recorded across the 4 states. A surge in AY clients seeking contraceptive services was observed in Ogun state between January 2019 and June 2020, and a surge was observed in Niger starting in June 2020. Since mid-2020, the number of AY clients seeking contraceptive services in Niger has continued to increase compared to the other 3 states. The highest number of clients in Niger state was recorded in May 2021. Niger and Ogun had the highest FP uptake compared to Edo and Plateau, which had less than 6,000 clients between December 2017 to September 2021 (Figure). The Figure shows how the trend for FP uptake for all states increased but at different rates and with little fluctuation.

**FIGURE.**
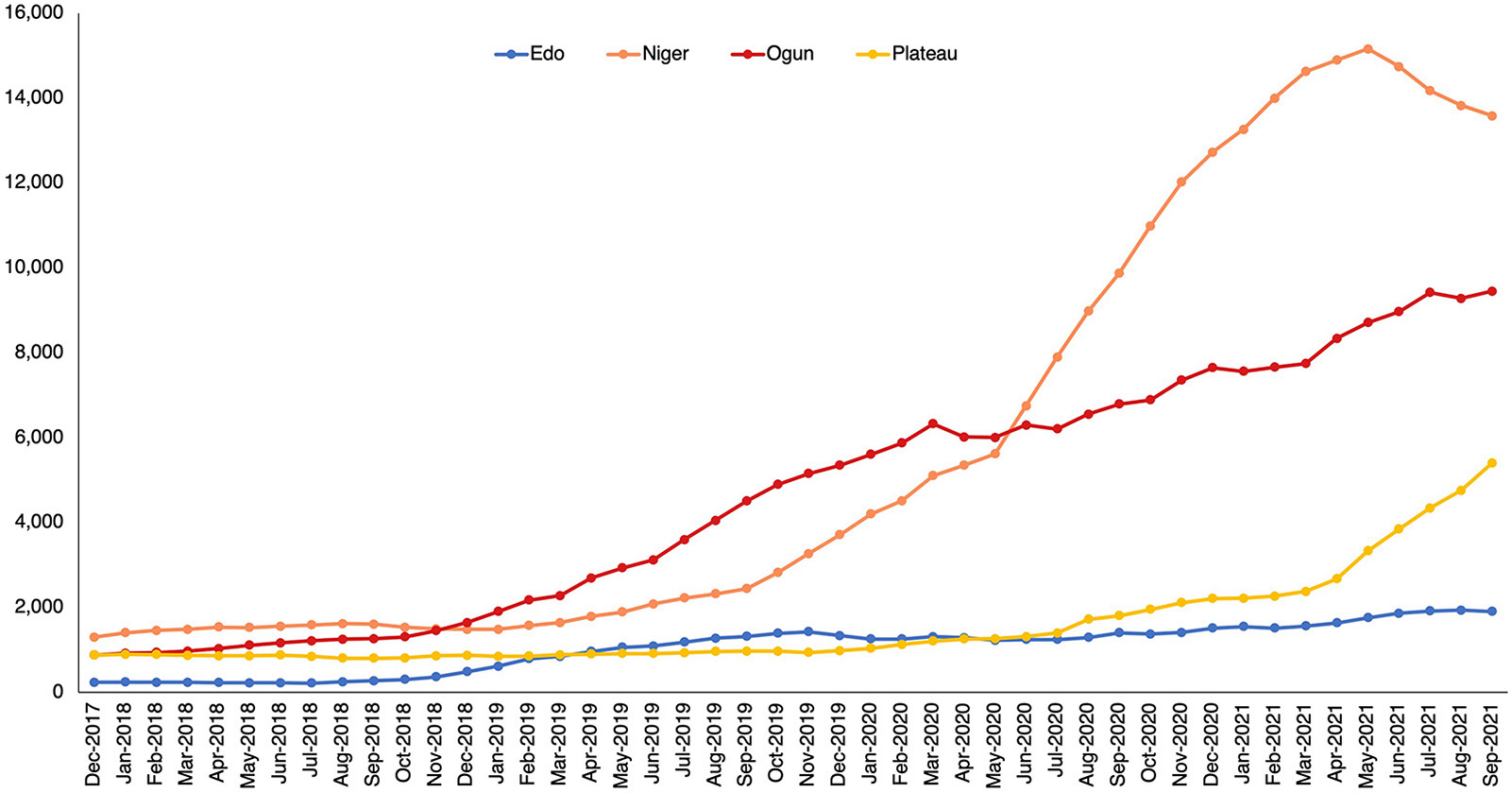
Family Planning Uptake by Adolescent and Youth Clients Across 130 High-Volume Facilities in Four Nigerian States

Table 3 describes how clients perceived the counseling and service provided at the health facilities. Of 739 clients across all the states, 96% stated that providers counseled them on the wide range of FP methods before they made their preferred choice.

**TABLE 3.** Clients’ Perception of Counseling and Service Provision Across Four Nigerian States

	**States**				
**Variables**	**Edo, %** **(n=105)**	**Niger, %** **(n=186)**	**Ogun, %** **(n=276)**	**Plateau, %** **(n=172)**	**Total, No. (%)** **(N=739)**
Told about family planning methods during counseling before choosing a method					
No	1.9	1.1	0	2.3	8 (1.1)
Yes	96.2	98.9	94.2	97.1	712 (96.3)
Refused to answer	1.9	0	5.8	0.6	19 (2.6)
Feel your choice of method was respected by the provider					
No	5.7	0.5	0.7	0	9 (1.5)
Yes	93.3	99.5	94.6	98.8	714 (96.5)
Refused to answer	1.0	0.0	4.7	1.2	16 (2.0)

Table 4 highlights whether clients reported service providers used SBC materials in the facilities that met the minimum quality standards. Almost 69% of clients surveyed said they were counseled with job aids, and 87% of the clients reported that they were provided with SBC materials. While more than 60% of respondents from the facilities that met the minimum quality standards of AYFHS in Niger, Ogun, and Plateau states reported being counseled with job aids, only 28% of clients in Edo state were counseled with job aids. The pattern is the same with the provision of pamphlets and brochures. More than 90% of respondents in the 3 states were provided with SBC materials on FP methods, while only 58% reported the same in Edo state.

**TABLE 4.** Provider’s Use of Social and Behavior Change Materials During Counseling Across Four Nigerian States

	**States**				
**Variables**	**Edo** **(n=105)**	**Niger** **(n=186)**	**Ogun** **(n=276)**	**Plateau** **(n=172)**	**Total, No. (%)(N=739)**
Clients were provided with pamphlets or brochures with information on family planning methods					
No	41.0	9.7	3.6	5.2	80 (10.8)
Yes	58.1	90.3	91.3	94.8	644 (87.1)
Refused to answer	0.9	0.0	5.1	0.0	15 (2.0)
Provider counseled with counseling job aids					
No	72.4	12.9	12.0	8.1	147 (19.9)
Yes	27.6	80.1	68.8	80.8	507 (68.6)
Sometimes	0.0	6.5	12.7	10.5	65 (8.8)
Refused to answer	0.0	0.5	6.5	0.6	20 (2.7)

Table 5 reports client satisfaction, perceptions, and feedback among those who visited a facility that met the minimum quality standards for AYFHS. Eighty percent of the total clients surveyed said they had sufficient time for consultation with the service provider. When disaggregated by state, 89% of clients from Niger and Plateau each said they had sufficient time to discuss with the provider during FP consultation visits to the facility compared with only 55% of clients from Edo state. Almost 9 of 10 clients in Niger and 8 of 10 clients in Plateau said they had sufficient time when attended to during their visit to the facility. Overall, almost 14% of them said they were sometimes asked to come back after adopting an FP method.

**TABLE 5.** Client Satisfaction, Perceptions, and Feedback on Family Planning Services in Four Nigerian States

	**States**				
	**Edo, %** **(n=105)**	**Niger, %** **(n=186)**	**Ogun, %** **(n=276)**	**Plateau, %** **(n=172)**	**Total, No. (%)** **(N=739)**
Clients have sufficient time for consultation with health care provider					
No	24.8	3.2	8.7	1.2	58 (7.8)
Yes	55.2	88.7	79.0	89.0	594 (80.4)
Sometimes	20.0	8.1	7.6	9.9	74 (10.0)
Refused to answer	0.0	0.0	4.7	0.0	13 (1.8)
Preferred contraceptive method always available when clients visit					
No	17.1	3.8	3.6	7.6	48 (6.5)
Yes	54.3	86.6	73.5	71.5	544 (73.6)
Sometimes	20.0	9.1	18.1	20.9	124 (16.8)
Refused to answer	8.6	0.5	4.7	0.0	23 (3.1)
Client satisfied with current contraceptive method					
No	6.7	1.6	2.2	0.6	17 (2.3)
Yes	81.0	96.8	90.2	97.7	682 (92.3)
Sometimes	9.5	1.6	1.8	1.2	20 (2.7)
Refused to answer	2.9	0.0	5.8	0.6	20 (2.7)
Client knew what to do when having contraceptive related side effect					
No	15.2	7.5	1.1	3.5	39 (5.3)
Yes	71.4	85.5	91.7	94.8	650 (88.0)
Sometimes	10.5	7.0	1.1	1.7	30 (4.1)
Refused to answer	2.9	0.0	6.2	0.0	20 (2.7)
Feel you are being treated respectfully and kindly by the provider					
No	21.9	1.1	0.4	0.0	26 (3.5)
Yes	64.8	92.5	90.6	98.3	659 (89.2)
Sometimes	13.3	6.5	1.8	1.7	34 (4.6)
Refused to answer	0.0	0.0	7.2	0.0	20 (2.7)
Feeling judged by staff					
No	62.9	90.9	90.2	98.3	653 (88.4)
Yes	14.3	6.5	2.2	0.6	34 (4.6)
Sometimes	20.9	2.2	1.1	1.2	31 (4.2)
Refused to answer	1.9	0.5	6.5	0.0	21 (2.8)
Clients would like to visit the facility again or refer a friend/family member					
No	15.2	0.0	4.0	3.5	33 (4.5)
Yes	81.0	99.5	90.2	95.9	684 (92.6)
Refused to answer	3.8	0.5	5.8	0.6	22 (3.0)
Reasons why clients would visit facility/refer others					
Good quality of care received	35.2	40.3	39.5	33.7	279 (37.7)
Good attitude of health personnel	16.2	43.6	21.4	29.7	208 (28.1)
Right to make FP choice(s)	14.3	8.6	5.8	15.1	73 (9.9)
FP commodity in stock	7.6	5.4	5.1	16.9	61 (8.3)
Refused to answer	26.7	2.2	28.3	4.7	118 (16.0)

Abbreviation: FP, family planning.

About 72% of the clients across the facilities said that their preferred contraceptive method is always available whenever they go to the facility for FP. Approximately 93% of the clients surveyed were satisfied with their current contraceptive methods.

When clients were asked whether they knew what to do when they had side effects due to the uptake of the method they chose, 88% of them reported affirmatively. Nine of 10 clients in Niger, Ogun, and Plateau said they were knowledgeable about what to do when they experienced any side effects attributed to the FP method that they were using. Only 7 of 10 clients in Edo reported they knew what to do when experiencing a side effect.

In addition, 89% of clients across the states felt that the service provider treated them with respect and kindness and took their opinion into consideration. However, only 6 in 10 clients in Edo state reported they were treated with respect. Additionally, almost 18% of Edo clients that visited a facility for FP services did not have the opportunity to ask questions or clarify any doubts about FP during consultation. Furthermore, Edo state had the highest proportion of clients (14%) that felt they were judged by the staff when they visited the facility compared with the other states, ranging from 0.6% from Plateau clients to 6.5% in Niger.

## DISCUSSION

Repeated rounds of the AYFPSS tool-driven QA process showed improvements in the quality of AYFHS provided to AY at the 130 HVS (Table 1). We found a consistent increase in the percentage of facilities in the scores between baseline, round 1, and round 2 of the assessment, reflected in improvements in the scores. Plateau, Ogun, and Edo states showed great improvements as 85%, 100%, and 70% of the selected HVS in their state, respectively, met the minimum quality standard at round 2 from a baseline of 0%. At baseline, Niger had 48% of facilities meet the minimum, but after rounds 1 and 2, 100% of facilities met the minimum standard.

These results show the relevance of the WHO quality AYFHS checklist. The systematic administration of the QA tool presented the facilities’ strengths and weaknesses in each of the domains and facilitated the development of tailored PIPs to be addressed within a given time frame that led to subsequent QI of AYFHS across 130 health facilities at different time periods. The findings also confirm that adapting this regional tool within an existing government supportive supervision health system can facilitate improvements in the quality of AYFHS and an increase in the associated uptake of modern contraceptive services by AY in combination with community-level demand-generating activities in supported states.^7^

The systematic administration of the QA tool highlighted facilities’ strengths and weaknesses in each domain and facilitated the development of tailored PIPs that led to subsequent QI of AYFHS across 130 health facilities.

While TCI supported the SMOH and SPHCDA to develop SBC materials informed by data and trained social mobilizers and providers to use these materials to generate demand for contraceptive uptake by AY populations, we were unable to assess the relative contributions of facility-level QI processes vis-à-vis the community-level demand generation activities toward increases in contraceptive uptake by AY. However, we know from Denno et al. that to promote access and uptake of AYFHS, adolescent-friendly facility-based approaches must be combined with activities that seek to improve community acceptance and demand generation, which is what we did.^6^ Our results highlight that our combined approach, anchored in the regular administration of the QA checklist at scale, improved the quality of AYFHS and contributed to the increase in contraceptive uptake by AY (Table 2).

The application of the AYFPSS and its associated QI processes showed improved quality of AYFHS and increased uptake in Ogun, Niger, and Plateau states as high as 195% to 536%. The FP uptake was lowest in Edo at 54%. In addition, the fewest number of HVS met the standards of quality AYFHS in Edo state. This difference could be linked to the length of the AYSRH program implementation period, which was longer in Ogun and Niger (3 years from September 2018 to September 2021) compared to less than 2 years in Edo state (from November 2019 to September 2021). Moreover, TCI had not previously supported Edo state’s FP program nor had any other implementing partner in recent years, but this was not the case in the other 3 states. However, Edo state’s results reaffirm, even in its early stages, that improving contraceptive demand and uptake by AY populations can be influenced through a combination of facility-level QI interventions and increased community-level demand generation.

We noted differences in client volume between the 4 states (Table 2). Plateau state had a lower client volume at round 2 assessment compared to Ogun state, although 100% of Plateau state’s HVS scored in the green compared to 85% of Ogun state’s HVS. This is likely a result of the difference in the number of HVS being compared. Plateau state only included 25 HVS, while Ogun included almost double that number with 47 HVS; therefore, the number of clients will differ.

Findings from this program case study show that it is possible to integrate an AYFPSS QA tool and its associated QI processes within an existing health system’s supportive supervision checklist, with the relevant support for enhanced capacity to administer, analyze, and translate the findings into action through the PIPs. Moreover, it appears that QI processes seemed to be more effective in states that had more robust existing health system supportive supervision structures than states with weaker or absent health system supportive supervision structures. Niger, Plateau, and Ogun states, where TCI had previously supported their FP programs, experienced quick and consistent rises in QI from baseline to round 2 compared to Edo state, where the health system had no existing FPSS before the AYSRH interventions.

QI processes seemed to be more effective in states that had more robust existing health system supportive supervision structures than states with weaker or absent health system supportive supervision structures.

Based on these results, further analysis of corresponding factors that influence demand and uptake of quality contraceptive services by AY should be undertaken while improving the quality of AYFHS through integrated supportive supervision. It would be interesting to see if the trend in contraceptive uptake continues in a positive, linear direction in Edo state in the future. Some questions remain. Will additional time be needed in Edo to ensure the implementation of a sufficient dose of the required resources to address QI issues like provider trainings and coaching, securing commodities, and procurement of equipment? And does there need to be sufficient exposure and additional time for demand-generation interventions to create an enabling and informed environment that would impact quality and uptake? In addition, an in-depth analysis of the length of time and frequency in the administration of the QA tool itself would be helpful in validating the tool and associated processes.

Although AYFHS is 1 of the interventions recommended by the WHO to address health system barriers that facilitate accessible, acceptable, equitable, appropriate, and effective quality of service,^3^ it is a challenge to ensure consistent and sustainable QI in doing so. The AYFPSS process could help facilitate improvements in AYFHS using existing health system supportive supervision structures of the Ministry of Health and government health agencies responsible for regulating the quality of health care. The simplified checklist accompanied by capacity strengthening in the form of coaching, whole-site orientation, and on-the-job training can positively impact the quality of AYFHS, as supported by client exit interview responses that reported satisfaction with services received from green facilities. Unfortunately, the lack of client feedback from poorer-performing facilities (scoring yellow and red) did not allow a comparison of client perceptions of poorer service quality.

Client exit interviews serve as an important accountability mechanism. And yet, the current AYFPSS does not include feedback from AY clients even though the original WHO-SEARO tool does. In fact, the WHO-SEARO tool also includes a domain for (community-level) demand generation as an essential domain for QA. In particular, the findings from the client exit interviews suggest that the AYFPSS tool can be improved and that there is still work to be done in strengthening providers’ training. For example, among those facilities that met the highest standards of quality in Edo, clients still reported feeling judged (14%) and not knowing what to do in the case of experiencing a side effect (30%). As a result, we recommend that the application of the AYFPSS and its accompanying processes should not be seen as a one-off intervention but built into the supportive supervision system to ensure ongoing behavior change among providers and system-level improvements to ensure adolescent-responsive contraceptive services that are available long term.

To enhance the sustainability of the AYFPSS tool and its accompanying QI processes, skilled personnel at the state level need to be available and have a written requirement in their job descriptions to administer the AYFPSS tool as part of supportive supervision. This will ensure that the AYFPSS tool and its accompanying QI processes become routine practice. Furthermore, the translation of the findings to remedial plans (i.e., PIPs) that are then implemented and monitored with repeat assessments should be undertaken to facilitate the improved quality of AYFHS. This improved quality AYFHS in health facilities should be complemented with tailored AY demand-generation activities at the community level to mitigate the sociocultural barriers prohibiting AY from accessing the available quality AYFHS in the facilities.

### Limitations

There are some limitations to this study and the monitoring of the AYFPSS checklist and subsequent QI processes. The client exit interview was not factored into the original plan of implementation and was later included to get client feedback on improvements in quality as reported by the provider perspective, which was captured in the AYFPSS. Given that the adapted AYFPSS did not include client feedback, though it was 1 of the domains of the WHO-SEARO tool, it was considered important to obtain their perspectives via client exit interviews. However, since these steps were factored in toward the end of program implementation, there is no baseline on AY clients’ perceptions of services before a QI approach with the application of the AYFPSS. Furthermore, client exit interviews were only conducted at the end of round 2 and with only clients from facilities that scored green. To objectively answer the second programmatic question posed in this case study, the client exit interviews would need to have been conducted with clients at all HVS, including those that scored yellow and red, starting at baseline and at the end of rounds 1 and 2 to potentially track improved client satisfaction with improvements in the quality of service provision. In addition, this program case study lacks data from a comparison site on levels of quality and AY client volume before implementation and at rounds 1 and 2. This limits the ability to ascribe causality to draw conclusions about the impact of the intervention, including facility-level application of the QA tool, the follow-up PIPs and QI processes, and the contribution of community-level demand generation in leading to increases in AY client volume. Lastly, data for this article are limited to adolescents aged 15–19 years and youth aged 20–24 years because these are the age bands for age disaggregation that the Government of Nigeria is piloting for inclusion in its HMIS. Moreover, during the period of this case study, the piloting of the “youth age band” for the HMIS had not commenced in the 4 states, necessitating a time-consuming extra step of data collation from the facility registers to document the numbers of clients aged 15–19 years and 20–24 years who received contraceptives.

## CONCLUSION

This program case study demonstrates that improving the quality of AYFHS is possible within the existing health system’s supportive supervision tools and processes. The findings are timely as there is growing recognition of the need to make existing health services adolescent-responsive and youth-friendly instead of having stand-alone service delivery models for contraceptive services for AY. In addition, integrating the WHO SEARO AYFHS checklist and national minimum standards for AYFHS into existing supportive supervision processes at the subnational level is doable. TCI’s approach to working in true partnership with state and LGA staff to enhance an existing tool and ensure its simplicity in design and application helped ensure its relevance and acceptance for use. Other features of the intervention’s success were leveraging supportive supervision and supporting the training and orientation of existing trained public health system staff, which were important factors to QI of AYFHS. Specific focus on improving community-level demand generation further supported the uptake of contraceptives by AY. The routine use of the AYFPSS checklist helped identify weaknesses at the facilities, which were addressed through targeted coaching and on-the-job training interventions, leading to improved quality AYFHS as noted in subsequent QA rounds. We believe that this approach can be readily adapted by other health program managers at state and LGA levels, donors, and implementing partners interested in supporting and improving quality AYFHS by strengthening government systems to drive implementation.

## Supplementary Material

GHSP-D-22-00169-supplement.pdf

## Notes

**Peer Reviewed**

**First published online:** April 4, 2023.
