# Supplementary material for: Improving the Quality of Adolescent and Youth-Friendly Health Services Through Integrated Supportive Supervision in Four Nigerian States
Source: Glob Health Sci Pract. 2024 May 21;12(Suppl 2):e2200169. doi: 10.9745/GHSP-D-22-00169 (PMC11111107; doi:10.9745/GHSP-D-22-00169)
Supplement: GHSP-D-22-00169-supplement.pdf [file GHSP-D-22-00169-supplement.pdf]

Supplement to: Akila D, Oluwasegun A, Bose K, Omotoso O, Adefila A, Mwaikambo L. Improving quality adolescent- and youth-friendly health service through integrated supportive supervision in 4 Nigerian states. Glob Health Sci Pract. 2023;11(Suppl 1):e2200169. https://doi.org/10.9745/GHSP-D-22-00169

Get it together for a brighter future

KNOW. TALK. GO.

Support Childbirth Spacing

www.tciurbanhealth.org | www.tciurbanhealth.org/tci-university

BE WISE!

See Your Health Provider.
Make a Choice Now!

Modern Family Planning Methods: Safe and Trusted

Get it together for a brighter future
KNOW. TALK. GO.
Support Family Planning

THE CHALLENGE INITIATIVE tci
NIGERIA

TCI 2022

FAMILY PLANNING / CHILDBIRTH SPACING METHODS

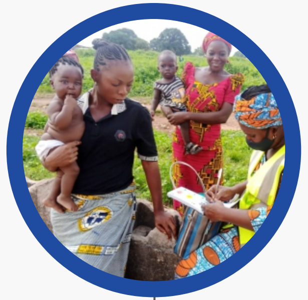

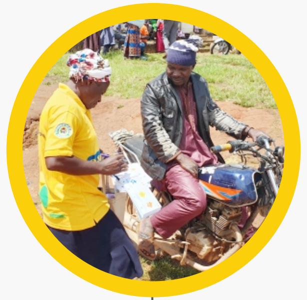

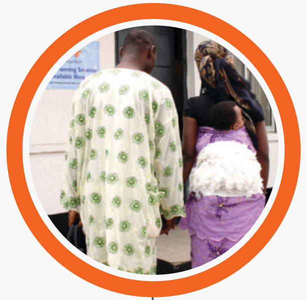

KNOW the facts about family planning

TALK to your partner about family planning

GO for family planning

The right method is the one that suits you

Supplement to: Akila D, Oluwasegun A, Bose K, Omotoso O, Adefila A, Mwaikambo L. Improving quality adolescent- and youth-friendly health service (AYFHS) through integrated supportive supervision in 4 Nigerian states.
Glob Health Sci Pract. 2023;11(Suppl 1):e2200169. https://doi.org/10.9745/GHSP-D-22-00169

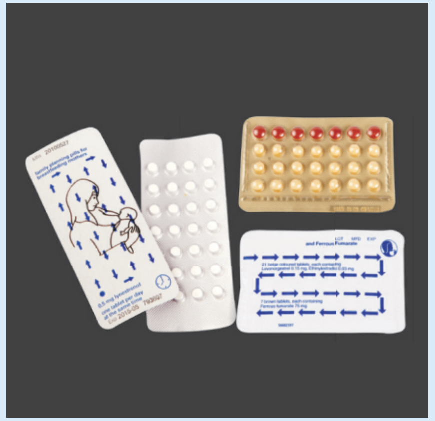

Pill

- Effective short-acting method that is taken everyday
- Safe for women of any age, including women who have never had a baby
- The mini-pill is safe for breastfeeding mothers.

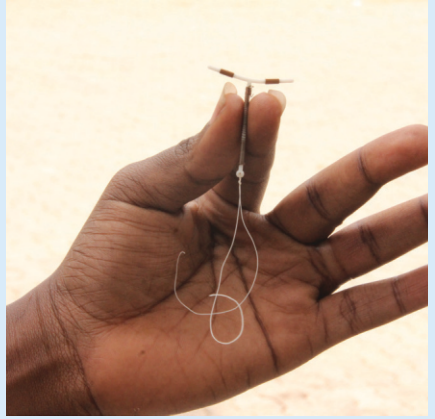

IUD

- Effective long-acting method that lasts 5-10 years
- Safe for women of any age, including women who have never had a baby
- Can use within 48 hours of childbirth
- Safe for breastfeeding mothers.

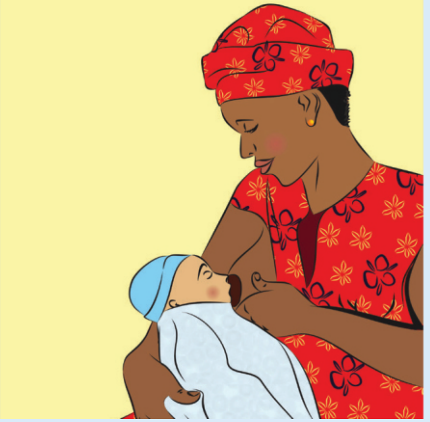

Exclusive Breastfeeding Method (LAM)

- Effective post-partum method when women meet all three criteria:
  - Are breastfeeding exclusively (day and night)
  - Have an infant younger than 6 months old
  - Do not have menstrual bleeding.

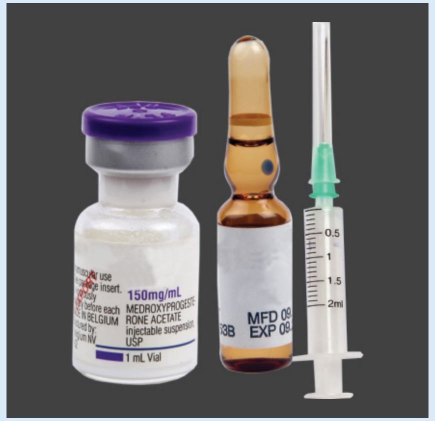

Injectable

- Effective short-acting method that lasts 2 or 3 months
- Safe for women of any age, including women who have never had a baby
- Safe for breastfeeding mothers with a baby more than 6 weeks old.

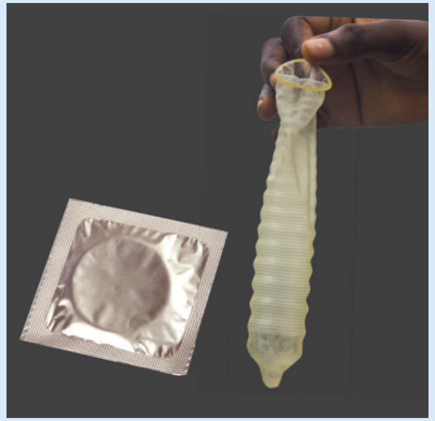

Male Condom

- Effective short-acting method that is used at the time of sex
- When used correctly at every time, it:
  - Prevents the female partner from getting pregnant
  - Prevents Sexually Transmitted Infections (STIs), including HIV/AIDS.

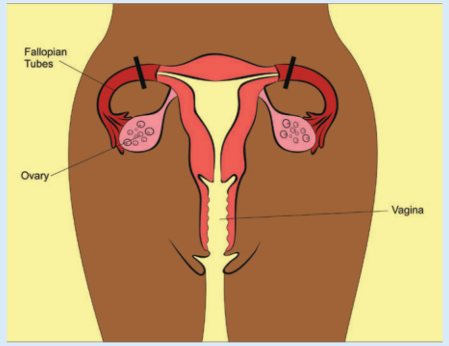

Tubal Ligation

- Effective permanent method for women who do not wish to get pregnant again.

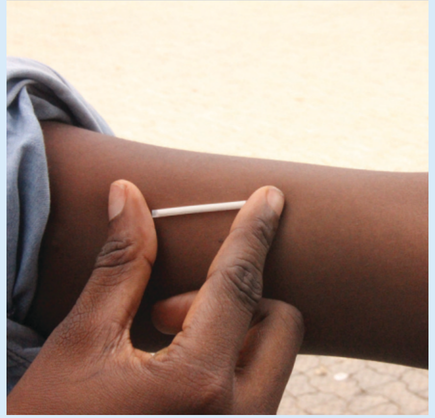

Implant

- Effective long-acting method that lasts 3-5 years
- Safe for women of any age, including women who have never had a baby
- Safe for breastfeeding mothers.

© 2000 Center for Communication Programs,
Courtesy of Photoshare

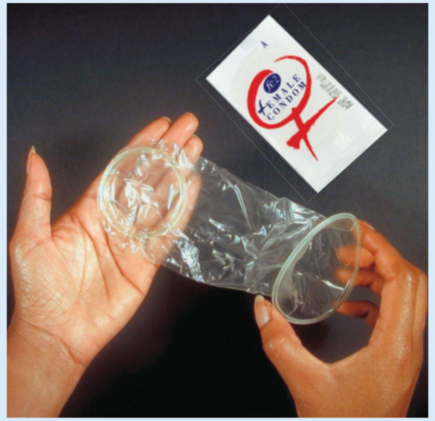

Female Condom

- Effective short-acting method that is used at the time of sex
- When used correctly at every time, it:
  - Prevents pregnancy
  - Prevents Sexually Transmitted Infections (STIs), including HIV/AIDS
  - is safe for breastfeeding mothers.

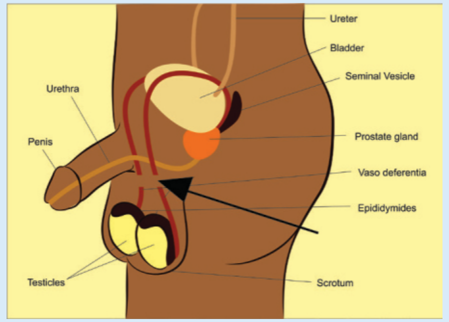

Vasectomy

- Effective permanent method for men who do not want their partners to get pregnant again.

Supplement to: Akila D, Oluwasegun A, Bose K, Omotoso O, Adefila A, Mwaikambo L. Improving quality adolescent- and youth-friendly health service through integrated supportive supervision in 4 Nigerian states. *Glob Health Sci Pract*. 2023;11(Suppl 1):e2200169. https://doi.org/10.9745/GHSP-D-22-00169

SUPPLEMENT. THE ADOLESCENT AND YOUTH-FRIENDLY FAMILY PLANNING SUPPORTIVE SUPERVISION CHECKLIST DOMAINS

**Service provision**	Service providers were interviewed, and registers were checked for SRH services provided, service fees for contraceptive methods provided (condoms, hormonal pills, emergency pills, other methods), and other services including referrals from in-reaches and outreach records.
**Facility characteristics**	Facilities were visited to check for the following: signboards with clinic information, such as operating hours and services offered, confidentiality policy on display, consultation/examination room that ensures auditory and visual privacy, registers with age-disaggregated records for AY clients kept under lock to ensure confidentiality, and stock of commodities and supplies.
**Capacity of service providers**	The main service providers in the HVS were interviewed to assess their training needs, trainings received, including trainings on general FP service provision, interpersonal communication and counseling (IPCC) skills including overcoming provider bias, commodity logistics management system (CLMS), short-acting reversible contraceptives (SARC), long-acting reversible contraceptives (LARC), and whole site orientation.
**Information provision**	The AYFPSS also assessed availability of SBC materials on AYFHS at the facility for clients to see, dissemination of AYFHS information to and from community members and AY (with records of activities and referrals from demand generation activities by community-based organizations (CBOs) and volunteers from the community), and dissemination of AYFHS/AYRSH information to adolescents in the community (with records of activities with adolescent groups).
**Data records**	Availability of facility register and records with documentation of number of AY attending, disaggregated by sex and age 15-19, 20-24, and 25 above was checked and clearly stating the different methods provided/obtained.
